# Epidemiology of Hepatitis E in 2017 in Bavaria, Germany

**DOI:** 10.1007/s12560-021-09474-0

**Published:** 2021-04-26

**Authors:** K. Hriskova, D. Marosevic, A. Belting, J. J. Wenzel, A. Carl, K. Katz

**Affiliations:** 1 Institute for Medical Information Processing, Biometry and Epidemiology - IBE, LMU Munich, Marchioninistr. 15, 81377 Munich, Germany; 2 Pettenkofer School of Public Health, Munich, Germany; 3 Bavarian Public Health and Food Safety Authority (LGL), Veterinärstraße 2, 85764 Oberschleißheim, Germany; 4 National Consultant Laboratory for HAV and HEV, Institute of Clinical Microbiology and Hygiene, University Medical Centre Regensburg, Franz-Josef-Strauß-Allee 11, 93053 Regensburg, Germany

**Keywords:** Hepatitis E, HEV, Genotype 3, Risk factors, Transmission

## Abstract

In the last decade, the number of reported hepatitis E virus (HEV) infections in Germany, including Bavaria, has continued to rise. In order to identify risk factors associated with HEV infection, we investigated notified hepatitis E cases from Bavaria during 2017. The project “Intensified Hepatitis E Surveillance in Bavaria” included interviews with questionnaires, collection and genotyping of stool, serum and food samples. In addition, certain risk factors were examined in a sample comparison with healthy population using univariable analysis and logistic regression. In total, 135 hepatitis E cases from Bavaria were included in the analysis. Mean age for women was 46 (range 20–74) years and 47.5 (range 20–85) for men. 56 of the cases (41.5%) were asymptomatic. Among the symptomatic cases, both men and women were equally affected with symptoms like fever (16.3%), jaundice (18.8%) and upper abdominal pain (28.2%). 145 human samples (serum, stool) and 6 food samples were collected. 15.9% of the human samples (*n* = 23) were positive for HEV RNA by reverse-transcription quantitative real-time PCR (RT-qPCR). Identified risk factors significantly associated with hepatitis E were sausage consumption with odds ratio 9.6 (CI 1.3–70.1), fish with OR 2.2 (CI 1.1–4.4) and cat ownership with OR 1.9 (CI 1.3–3.0) in multivariable analyses. Further investigation is needed to confirm the role of fish in HEV transmission. Autochthonous HEV genotype 3 is prevalent in Bavaria and there could be more transmission routes contributing to the spread of HEV than previously known. Undercooked meat, offal, sausages, fish, shellfish and contact with animals and pets are possible sources for infection.

## Introduction

Hepatitis E virus (HEV) is a non-enveloped, single-stranded RNA virus that belongs to the Hepeviridae family and causes infectious inflammation of the liver. Human pathogenic HEV can be classified in four major genotypes (HEV 1–4), that can further be classified into subgenotypes. Genotypes 1, 2, 3 and 4 count seven, two, fourteen and nine subgenotypes, respectively (Smith et al., 2020). The clinical manifestation of an HEV infection varies in severity from subclinical to fulminant, probably, depending on the genotype and host factors (e.g. immune constitution). Genotypes 1 and 2 are endemic in developing countries in Asia and Africa, as well as in Mexico. They are restricted to humans, mostly transmitted through the faecal-oral route and causing major outbreaks in tropical and subtropical regions (Khuroo and Khuroo, 2016; Rein et al., 2012). Genotype 3 has been found mainly in European countries, USA and Japan, while genotype 4 is mostly spread in Asia (China, Japan and India) (Pavio et al., 2010). Genotypes 3 and 4 are zoonoses and have been detected in several animal species (e.g. pigs, wild boar, deer and rabbits) (Meng, 2013) and humans. These HEV genotypes can cause sporadic, self-limiting disease with clinical symptoms like vomiting, uncolored stools, darkened urine and jaundice (Nan et al., 2017). According to the German Infection Protection Act (IfSG) clinical suspicion, illness or death from acute viral hepatitis and/-or the direct or indirect detection of hepatitis E virus must be notified.

Since the introduction of IfSG in 2001, until 2014 the HEV incidence per 100.000 inhabitants in Germany was below 1. In recent years, however, the number of reported hepatitis E cases has substantially increased. In Bavaria and also nationwide (RKI, 2018), the number of new hepatitis E cases almost doubled in 2017 compared to 2016 (LGL, 2019). It is uncertain whether the growing number of reported cases is due to an actual increase in the number of infected humans or due to a diagnostic and notification biases. Over the last few years, the disease has come into sharp focus as it is no longer perceived only as a travel associated disease. Similar to other European countries, the autochthonous genotype in Germany is the genotype 3. The prevalence of antibodies against HEV in the adult population of Germany is 16.8% (about 5% in people under 30 years of age, up to 25% in those over 60 years of age) (Faber et al., 2012). Eating raw or uncooked meat, offal and meat products (e.g. sausages) were considered as a risk factor for hepatitis E in humans (Wichmann et al., 2008; Szabo et al., 2015; Faber et al., 2018; Said et al., 2017; Meng, 2010). Other reported risk factors associated with hepatitis E are the consumption of shellfish ( e.g. oysters, mussels) (Grodzki et al., 2014; O’Hara et al., 2018; Said et al., 2009; Mesquita et al., 2016) and the contact with waste waters (Clemente-Casares, 2016). Bavaria is a federal state with a high density of pigs and high seroprevalence for HEV in pig herds. On average 51.4% of tested pig sera samples in Southern Germany (Bavaria and Baden-Wuerttemberg) exhibited anti-HEV antibodies (Krumbholz et al., 2013). Direct contact with pigs is considered a risk factor for hepatitis E (Chaussade et al., 2013; Krumbholz et al., 2014). Furthermore, IgM and IgG antibodies against HEV were detected further up the food chain in serum and meat juice samples from Bavarian slaughterhouses (Wacheck et al., 2012). In addition, 4% of pork livers purchased from butchers in Regensburg, Bavaria, had detectable levels of HEV RNA (Wenzel et al., 2011). Based on the above evidence and the increasing number of new infections in Bavaria, intensified hepatitis E surveillance was conducted in 2017. The project had three major objectives: 1. to describe the population diagnosed with hepatitis E in Bavaria, 2. to identify the most common subgenotypes of HEV circulating in Bavaria, 3. attempt to identify the risk factors associated with transmission of HEV genotype 3.

## Materials and Methods

### Data Collection of Hepatitis E Cases

**Fig. 1:**
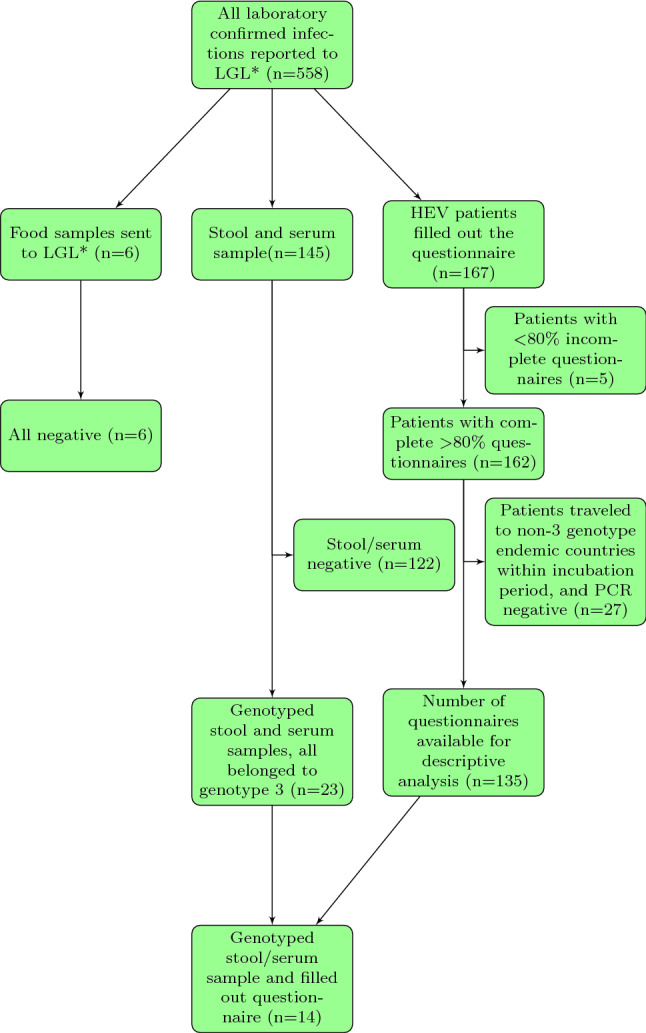
Study population. $^{*}$Bavarian Public Health and Food Safety Authority

The project “Intensified Hepatitis E Surveillance in Bavaria” was conducted from the third calendar week of 2017 until the fourth calendar week of 2018. The project included a specially designed questionnaire, collection of stool or serum samples from patients and food samples if available. A total of 558^1^ hepatitis E cases were reported to the Local Health Authorities (LHA) during the study period. The diagnostic methods for these 558 cases were IgM blood (*n* = 530), PCR blood (*n* = 46) and PCR stool (*n* = 15), where some cases were diagnosed by two methods. The participating health authorities informed the reported cases about the study and invited them to join the study. The participation in the study was anonymous and voluntary. Written consent was obtained from all recruited participants. Stool samples were analysed at the National Consultant Laboratory for Hepatitis A and Hepatitis E Virus, Institute of Clinical Microbiology and Hygiene, Regensburg. If available, food samples from patients’ households, suspected to be risk factor for HEV, were collected and analysed at the Laboratory for Food Virology of the Bavarian Public Health and Food Safety Authority (LGL) in Erlangen. Inclusion criteria for descriptive analysis of HEV cases were notified hepatitis E cases with laboratory confirmed HEV infection (IgM or PCR positive) with at least 80% completed questionnaire and no travel within the incubation period to HEV genotype 1, 2 or 4 endemic countries, according to a resent review article Perez-Gracia et al (2015) (Fig. 1). Plausibility check was performed to verify answer consistency. All available sequenced serum and stool samples were included in the subgenotype analysis irrespective of the completeness of the questionnaire.

### Laboratory Confirmation and Genotyping of HEV

#### RNA Extraction and Molecular Detection

Nucleic acid isolation from human specimens was performed from $200\,\upmu \hbox {l}$ serum or stool suspension on an EZ1 Advanced XL workstation using the EZ1 Virus Mini Kit v2.0 (Qiagen, Hilden, Germany). Eluted nucleic acid was analysed by RT-qPCR as previously described (Wenzel et al., 2011). The lower limit of detection (95% LoD) for HEV RNA was 1200 IU/ml.

#### Sequencing of HEV RNA

HEV RT-qPCR positive samples were further characterized by amplicon sequencing. The initial amplification was performed by using specific primers for a fragment of HEV-ORF1 (418 nt, FJ705359 pos. 54-471) (Wenzel et al., 2011). Moreover, a fragment of the RNA-dependent RNA polymerase (RdRp, 470 nt, FJ705359 pos. 4181-4650) was amplified according to a published protocol (Johne et al., 2010). The nested PCRs were performed with specific primers for HEV-ORF1 (286 nt, FJ705359 pos. 102-387) and RdRp (332 nt, FJ705359 pos. 4286-4617), respectively. The PCR products were purified by using QIAquick columns (Qiagen, Hilden, Germany) and sequenced in both directions with the nested PCR primers. Nucleotide sequences of amplicons were determined by using the BigDye Terminator cycle sequencing kit (Applied Biosystems) and separated on a model 3730xl genetic analyzer (Applied Biosystems, Waltham, USA). Nucleotide sequences of PCR products were analysed by using CodonCode Aligner software (http://codon-code.com/).

#### Phylogenetic Analyses

A maximum likelihood phylogenetic consensus tree for partial HEV-ORF1 and RdRp nucleotide sequences was inferred using RAxML version 8.2.11 (available at: https://sco.h-its.org/exelixis/web/software/raxml). The sequences derived from the patients’ samples were put into context with the published reference sequences for HEV subtypes which are currently known to infect humans (Smith et al., 2020).

### Population from the DEGS1 Study

In addition to the recruited HEV cases, a dataset from the Robert Koch Institute (RKI, Berlin) was used in order to compare alimentary habits of Bavarian hepatitis E cases in 2017 with the general healthy adult population of Germany participating in the RKI survey. This survey was conducted between November 2008 and December 2011 called “Study on adult health in Germany” (DEGS1) (DEGS1, 2015). Details about this study are described elsewhere (Scheidt-Nave et al., 2012; Gößwald et al., 2012). Briefly, the study included randomly selected individuals living in Germany between 18 and 79 years, recruited randomly from residents’ registration offices. The data collection included interviews with detailed Food Frequency Questionnaire (FFQ) and additional measurements and tests carried out in participating health care facilities. A total of 7.009 participants completed the FFQ. Inclusion criteria for our study included living in western federal states as federal district per participant was not available ($\textit{n}=4.785$) and no diagnosis of hepatitis in the past (hepatitis A, B, C, D, E or unknown type) ($\textit{n}=4.512$).

### Statistical Methods

Descriptive analysis for Bavaria’s hepatitis E cases was performed and the absolute and relative frequencies were reported. *T* test and ANOVA were used to test if the differences in the mean age between women and men are significant. Chi Square Test was used to investigate the association between variables with two categories. In addition, the detailed questionnaire for HEV cases and the FFQ questionnaire completed by the DEGS participants contained 6 identical questions on dietary habits and 2 questions on pet husbandry. On the basis of these 8 questions the two populations, HEV cases ($\textit{n}=135$) and baseline population ($\textit{n}=4.512$), could be compared. A univariable analysis with a total of 8 variables was performed by calculating odds ratios (OR) and 95% confidence intervals (CIs). Thereafter a logistic regression was conducted. Considering the AIC as selection criterion for the best model, we decided to keep the full model (variables included see Table 2) as it resulted in the smallest AIC. In addition, we wanted our final model to include age and sex variables, regardless of significance, in order to take into account possible confounding factors. The odds ratios, adjusted for gender and age, were reported. The analysis was performed with SAS University Edition.

## Results

### Descriptive Analysis of Bavaria’s Hepatitis E cases

**Fig. 2:**
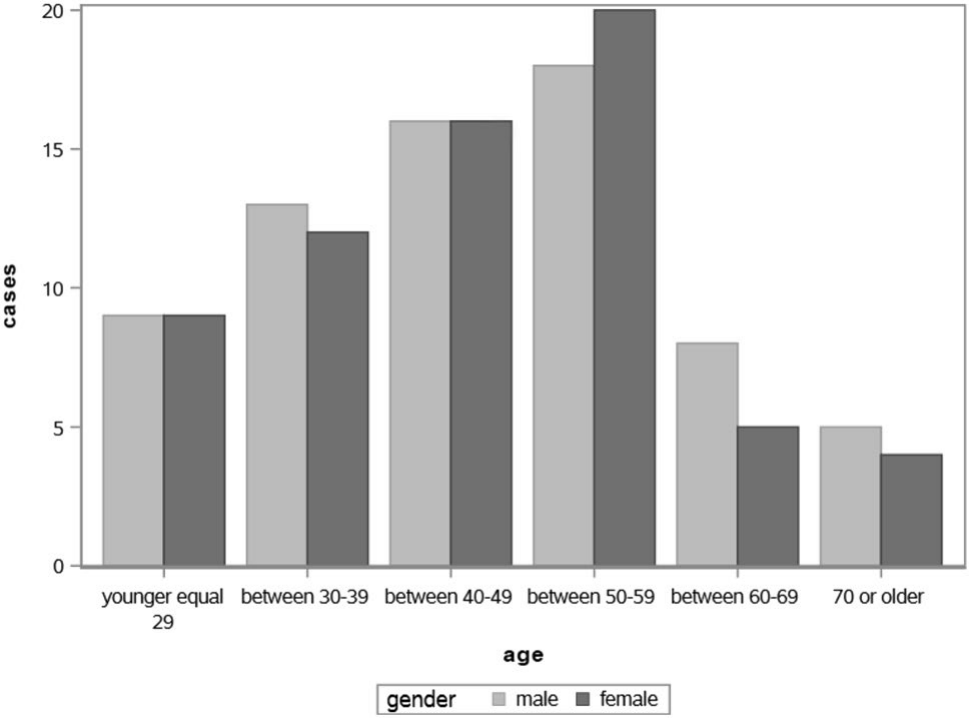
Gender and age distribution of the Bavarian hepatitis E cases

The questionnaire was filled out by 167 patients with hepatitis E, which corresponds to 30% of all reported HEV cases in Bavaria during the study period. A total of 5 questionnaires were filled out less than 80% and therefore excluded from further evaluation. (Fig.  1). Out of the remaining 162 patients, 35 had travelled abroad. Eight of them visited countries where genotype 3 is prevalent, during the relevant incubation period. 27 patients visited countries, where other or unknown HEV genotypes are endemic (e.g. Singapore, Nicaragua, Namibia) . Stool samples were available from 17 of these 27 patients, but HEV genotype 3 could not be detected by RT-qPCR. As our aim was to focus on risk factors associated with transmission of HEV genotype 3, these 27 were not considered in the further analysis. In total, 66 women and 69 men were included in the study. The mean age was 46 years for women (range 20–74 years) and 47.5 years for men (range 20–85 years). (Fig. 2). There was no significant difference in the mean age between women and men (*t* test and ANOVA).

**Table 1:** Descriptive analysis of hepatitis E cases

Variable	All cases ($\textit{n}=135$)	%
Symptoms & previous diseases	Yes	
Symptoms	79	58.5
Elevated liver enzymes	76	56.3
Upper abdominal pain	38	28.2
Icterus	24	17.8
Fever	22	16.3
Liver disease	21	15.6
Fatty liver	15	11.1
Immunosuppression	12	8.9
Liver infection	3	2.2
Hepatitis C	2	1.5
Blood transfusion	1	0.7
Liver cirrhosis	1	0.7
Meat consumption	Yes	
Pork on piece	126	94.7
Raw ham	120	89.6
Beef not fully cooked	68	51.5
Wild boar	35	26.1
Pig liver	33	24.6
Meat products consumption	Yes	
Salami	131	97.0
Cooked sausage	128	95.5
Liver pate	98	73.1
Other risk factor consumption	Yes	
Fish	123	91.8
Raw vegetable	121	90.3
Raw fish	40	30.1
Grill party	Yes	
Visited grill party	63	46.7
Consume pork/game	55	87.3
Prepared pork/game	44	69.8
Pets	Yes	
Cat	39	28.9
Dog	20	14.8
Rabbits	5	3.7
Other rodents	2	1.5
Direct contact to animals	Yes	
Live/work on a farm	8	5.9
Working in butcher shop	2	1.5
Water contact	Yes	
Wastewater	4	3.0
River/lake	3	2.2
Fountain	3	2.2

There were 79 (58%) cases with symptoms. The most frequent ones were: increased liver values (56%), upper abdominal pain (28%), jaundice (17%) and fever (16%). Other symptoms recorded by the patients were fatigue, itching, nausea, aching limbs, muscles and legs. No association between gender and the presence of symptoms (Chi Square Test) was detected. One patient reported having received a blood transfusion during the incubation period. Three patients had a chronic liver disease, and two of them reported Hepatitis C. Twenty-one patients reported other liver disease among which the most common was fatty liver disease ($\textit{n}=16$) (Table 1).

The specially designed questionnaire contains several items on the consumption of potential HEV risk food products such as various meat types, cold cuts and sausages. Four patients indicated to eat halal, one kosher and one vegetarian. The only person who said he was eating kosher also checked that he was eating halal. After the check for answer consistency there were no strictly vegetarian, no strictly kosher and three real halal eating patients. Notable is that 91% of the cases ($\textit{n}=123$) reported having consumed raw or poorly cooked meat or raw mussels or raw fish at least 1 to 3 times per month during the incubation period.

### Human and Food Samples

**Fig. 3:**
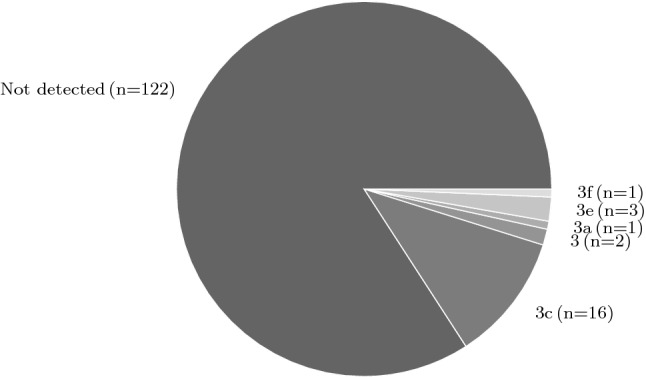
Detected subgenotypes

A total of 145 human samples (143 stools and 2 sera), were tested for the presence of HEV RNA. There were 39 patients that provided stool or serum samples but did not fill out the questionnaire. Nevertheless all available samples were included in the subgenotype analysis. Most of the human samples ($\textit{n}=122$) were negative for HEV RNA by RT-qPCR. A total of 21 stool samples and 2 serum samples were genotyped (GenBank accession no. HG998145-HG998188), 14 of them with an accompanying completed questionnaire. All samples were classified as HEV genotype 3 (Fig. 3). The most commonly found subgenotype was 3c ($\textit{n}=16$), followed by 3e ($\textit{n}=3$), 3a ($\textit{n}=1$), 3f ($\textit{n}=1$) and 3 not further specified ($\textit{n}=2$). Six food samples such as minced meat, “Kaminwurzen” (smoked pork sausages), beef meat and deer goulash were collected and tested. All were HEV RNA negative (Fig. 1).

### Comparison of the HEV cases and the DEGS1 population

In the DEGS population included in the study ($\textit{n}=4.512$), 48% of the participants were male ($\textit{n}=2.156$) and 52% female ($\textit{n}= 2.356$). The mean age overall was 57.2 range (24–88), with no significant difference between the sexes (ANOVA and *T* test). Thus the DEGS participants were considerably older than the hepatitis E cases reported in Bavaria in 2017 ($\hbox {average}\ \hbox {age} = 46.8\ \hbox {years}$). The difference in age between the two populations is also significant (*T* test), while no difference was observed regarding the sex of the participants (Chi-square test). In addition to age and gender, 8 risk factors were tested in the univariable analysis. Six of them were about nutrition habits and two risk factors considered the possession of dogs or cats as pets. The consumption of sausages (like salami, liver sausages, raw spread sausages), cooked fish (e.g. pollack, trout) were significantly associated with hepatitis E. (Table 2). Owning a cat is significantly associated with hepatitis E, as opposed to owning a dog. Other variables not significantly associated with hepatitis E infection were the consumption of meat (e.g. pig, wild boar, deer) and raw ham. The variable consumption of raw vegetable seems to be a significant protective factor for hepatitis E infection.

**Table 2:** Risk factors (*NA*—the effect of the variable is not significant in the Multivariable Analysis, **Corresponds to significance level 5%, ***Corresponds to significance level 1%)

Risk factors	Cases ($\textit{n} = 135$)		Baseline population ($\textit{n} = 4.512$)		Univariable analysis		Multivariable analysis	
Variable	Yes	%	Yes	%	Odds ratio	95% CI	Odds ratio	95% CI
Sausages consumption (no ham)	133	98.5	4.037	89.7	7.7***	1.9–31.1	9.6**	1.3–70.1
Cat possession	39	31.2	676	15.4	2.5***	1.7–3.7	1.9***	1.3–3.0
Fish consumption (cooked)	123	91.8	3.811	84.8	2.0**	1.1–3.7	2.2**	1.1-4.4
Raw vegetable consumption	121	90.3	4.245	95.3	0.5**	0.3–0.8	0.4**	0.2–0.8
Ham consumption	120	89.6	3.828	85.0	1.5	0.9–2.6	NA	
Meat consumption (no chicken, no sausages)	131	97.0	4.326	96.2	1.3	0.5–3.6	NA	
Dog possession	20	16.1	608	13.9	1.2	0.7–1.9	NA	
Vegetarian diet	1	0.8	185	4.1	0.2	0.0–1.3	NA	

## Discussion

### Bavarian Hepatitis E Cases and Risk Factors

During 2017 an intensified hepatitis E surveillance was conducted in Bavaria. This included a detailed questionnaire, stool and serum samples, and food investigations from notified hepatitis E cases. A total of 135 questionnaires, 145 human samples and 6 food samples were investigated in order to gain deeper understanding of the risk factors for HEV transmission. The recruited hepatitis E cases from Bavaria ($\textit{n}=135$) were compared with DEGS study population from West federal states in Germany ($\textit{n}=4.512$) by univariable and multivariable analyses.

Case reports and series from Europe suggest that HEV genotype 3 is more common in elderly men (Festa et al., 2014; Dalton et al., 2008; Chalupa et al., 2014; Saint-Jacques et al., 2016; Tarantino et al., 2016; Borgen et al., 2008). However, seroprevalence studies are not very conclusive. One study concluded that the older (30–39) participants and male participants were more often positive for anti-HEV IgG than younger (20–29) or female participants in southern Germany. Since the study comprised only young participants aged 20–39, it is unclear whether this trend holds true also among the older population (Mahrt et al., 2018). In another larger study, with adults aged 18–79 years from all federal states in Germany no gender difference in the HEV seroprevalence was reported, but an increase in seroprevalence with age. The authors put forward the idea that food frequently consumed by both sexes plays a major role as a channel of transmission (Faber et al., 2012). The authors of a systematic review suggested that the higher incidence of clinical cases in men indicates rather gender differences in the development of diseases or the use of laboratory tests and not difference in infection rates (Lewis et al., 2010). Our results are in line with Faber et al. (2012), since both male and female participants of the study were almost equally represented; however, both male and females were also equally affected with symptoms in our study and, therefore, not confirming the higher manifestation rates among males. However, we had a lower percentage of participation in the study from the population over 60. Among all cases reported in Bavaria during this period ($\textit{n}=558$), 27% were over 60 years old, while in our study, only 17% were over 60 year old. This could indicate that older people are unable or less motivated to complete the detailed questionnaire and therefore less likely to participate in the study.

#### Risk Factors Associated with Food Consumption

To the best of our knowledge no study in Europe investigated fish consumption as a risk factor for transmission of autochthonous Hepatitis E. HEV genotype 3 was detected in 32.2% (10 of 31) dolphins at the National Aquarium, Havana, Cuba. Dolphins generally feed on fish and squid. Infected animals or contaminated surface waters could be the source of infection (Villalba et al., 2017). Other seafood such as diverse shellfish—bivalve in Japan (Li et al., 2007), mussels in UK (Crossan et al., 2012), Spain (Mesquita et al., 2016), Italy (La Rosa et al., 2018) and 2.9% of shellfish purchased in local supermarkets in Scotland (O’Hara et al., 2018) have been tested positive for HEV genotype 3. An HEV outbreak on a cruise ship was linked to the consumption of shellfish on board (Said et al., 2009). In Italy, apart from the 2.6% HEV positive mussels, 12.8% seawater samples (La Rosa et al., 2018) and raw sewage and river samples (Iaconelli et al., 2015) were also tested positive for HEV genotype 3. Since HEV genotype 3 is an enteric pathogen in both human and diverse animal species, human and animal faeces could contaminate the sewage and coastal water. In countries with high farming density and shellfish production close to the shore, shellfish can bio-accumulate human and animal enteric viruses (Mesquita et al., 2016; Grodzki et al., 2014). As shellfish is often consumed raw or undercooked, the virus has not been deactivated and can pose a risk for HEV infection. Fish could also be consumed raw or cooked thoroughly; however, it is not known whether bio-accumulation, similar to shellfish, can happen in fish. The cutthroat trout virus (CTV), a virus initially isolated in 1988 shows between 29 and 49% genome sequence similarity to HEV genotypes 1–4, rat HEV and avian HEV. Since 2010, a related agent was described from related salmonid fish species. The presence of CTV was confirmed in different trout species (Johne et al., 2014; Smith et al., 2014). Recently, the International Committee on Taxonomy of Viruses (ICTV) gave a new classification for the family Hepeviridae. All HEV’s have been placed under Hepeviridae family and further classified under 2 genera namely Orthohepevirus, which included isolates from mammals and chicken and Piscihepevirus, containing the fish HEV (Purdy et al., 2017). However, the zoonotic potential of CTV has not yet been clarified and its influence on the epidemiology of human hepatitis E is largely unknown (Johne et al., 2014). Therefore, it is not clear whether we see the effect of this HEV-like virus as a risk factor for HEV infection in humans. Further research is needed to investigate whether fish consumption can be a risk factor for hepatitis E genotype 3.

In our study the sausages, like salami and liver sausages, were significantly associated with hepatitis E with OR 9.6 in multivariable analysis. This is in accordance with other studies from Germany (Faber et al., 2018; Szabo et al., 2015) and other European countries (Di Bartolo et al., 2015; Pavio et al., 2014; Berto et al., 2013; Di Bartolo et al., 2012; Mooij et al., 2018; Moor et al., 2018).

Hepatitis E RNA was detected in several studies in Europe in different food products such as figatelli (30–58%) (Colson et al., 2010; Pavio et al., 2014), pork liver sausages (6–29%) (Di Bartolo et al., 2012; Berto et al., 2012; Pavio et al., 2014; Szabo et al., 2015), quenelles (25%) and dried salted liver (3%) (Pavio et al., 2014). All HEV sequences obtained in these studies were of genotype 3.

However, the human HEV infection dose is unknown and it needs to be investigated whether the viral load in meat and ready-to-eat products is sufficient to infect humans. One experiment aiming to answer this question was done with pigs. Two out of three pigs were successfully infected with HEV genotype 3 after ingesting an inoculum of 10 mL with $10^{6}$ genome equivalents per mL (Andraud et al., 2013). Another study estimated that figatelli (pig liver sausage from France, commonly eaten uncooked) contains $10^{3}$–$10^{6}$ HEV RNA copies per slice (Colson et al., 2010). Figatelli had already been described as a risk factor for HEV (Capai et al., 2019) and had been identified as the cause of infection in case reports and series (Colson et al., 2010; Moal et al., 2012), suggesting that the infectious dose for humans is comparable to the experimentally proven infectious dose for pigs. In addition, the virus shows relatively high stability in order to be completely inactivated, the meat must be cooked at 71 degrees for 20 minutes (Barnaud et al., 2012). Therefore, there is a potential risk to public health consuming raw and undercooked products.

In our analysis the consumption of meat (no chicken and no sausages) was not a significant risk factor for HEV infection, as opposed to findings from other studies and case reports (Faber et al., 2018; Wichmann et al., 2008; Slot et al., 2017; Legrand-Abravanel et al., 2010; Riveiro-Barciela et al., 2015; Rivero-Juarez et al., 2017). This might be due to the fact, that the question was stated very general in DEGS questionnaire. Similarly, because no detailed information was available on pork and pork liver consumption in the questionnaire completed by the DEGS study population, it was not possible to analyse it separately, although both have been identified as risk factors in the literature (Wenzel et al., 2011; Lewis et al., 2010; Faber et al., 2018). Our study indicated that there was no significant association between consumption of raw ham and HEV infection. This is in line with the results from case-control study in Germany (Faber et al., 2018), but opposed to the findings from England and Wales (Said et al., 2017).

The consumption of raw vegetables resulted as some kind of protective factor for HEV infection. One might speculate that people who consume a lot of vegetables consume less meat. However, this is not conclusive and vegetable-based diet is not necessarily excluding a HEV infection, as HEV has already been detected in vegetables, ready-to-eat salads and irrigation water (Purpari et al., 2019; Terio et al., 2017; Kokkinos et al., 2017).

During the research period six food samples (minced meat, Kaminwurzen—smoked pork sausages, beef meat and deer goulash) from households of notified hepatitis E cases were available for laboratory testing. All were negative for HEV RNA. It is possible that these food items were not the source of infection, but other food that were no longer available for testing.

#### Risk Factor Pet Ownership

Our results suggest that possession of a cat is significantly associated with HEV infection as opposed to ownership of dogs. Both cats and dogs have been found seropositive for HEV in a study from Brandenburg, Germany (Dähnert et al., 2014). While another study from Germany found that the possession of pets was a protective factor for hepatitis E and cat ownership was the most protective factor (Wichmann et al., 2008). The authors doubted this and suggested that due to the frequent preparation of raw liver as cat food, a pre-existing immunity could exist due to a subclinical infection among cat owners. Similarly, it was described that cats fed with kitchen leftovers have a higher risk of HEV seropositivity than cats fed with commercial food, suggesting a common source for HEV infection for both animals and humans (Liang et al., 2014). A case report from Japan also described a patient infected with HEV genotype 4, whose cat was positive for HEV antibodies (Kuno et al., 2003). Rodent hunting by cats has been suggested as a possible source of infection (Mochizuki et al., 2006); however, the literature is not consistent as to whether rats are competent hosts of HEV genotype 3 (Shukla et al., 2011; Purcell et al., 2011; Johne et al., 2010; Lack et al., 2012).

Interestingly, in our study women with cat ownership have a higher risk (OR 2.7) for hepatitis E than men with cat ownership, and women in the age group over 70 years have an even higher risk (OR 9.6). It is tempting to hypothesize whether women living in a shared household are more likely to care for the pets (feeding and cleaning) and are therefore more exposed and have a higher risk of HEV infection. However, this hypothesis needs further investigation and fuels an interesting research aspect that should be considered in the design of future hepatitis E research studies.

### Limitations of the Study

The cases analysed in our study are from 2017 and no controls were recruited. The comparison is based on data from the DEGS study, which are only partially comparable. DEGS participants were recruited from 2008 till 2011. To check the comparability of the healthy population (DEGS study) and the cases, we looked for trends in the consumption behaviour of the German population. According to the National Nutrition Survey (NVS) II and the so-called NEMONIT study, there were no significant changes in the consumption of vegetables, meat/meat products and fish/fish products between 2005–2007 and 2012/2013 (Gose et al., 2016). We assume that this trend continued further. The DEGS Population is considered healthy, i.e. without HEV infection, we had no access to information about IgG or IgM tests in the available dataset, although a subset of the dataset was used and published for a HEV seroprevalence study (Faber et al., 2012). We have relied on the information provided by the participants in the questionnaire. Therefore, it is possible that, if there were some asymptomatic acute hepatitis E cases or cases with HEV IgG (had the disease in the past) in the DEGS population, the actual impact of the risk factors would be even greater if they were excluded. The results presented here must be interpreted carefully. Firstly, the cases and controls used for the statistical analysis were surveyed using different questionnaires, and although risk factors such as sausages, salami and liver sausages are in line with published research, fish consumption and cat ownership must be carefully interpreted and considered as possible risk factors in future studies and study designs.

## Data Availability

All data about the HEV cases are available from the corresponding author or from durdica.marosevic@lgl.bayern.de on reasonable request. The DEGS1 Dataset used to support the findings of this study can be requested upon application from the Research Data Centre (Robert Koch Institute, MF4)

## Footnotes

1 The number 558 include all HEV cases reported within the study period, from the third calendar week of 2017 until the fourth calendar week of 2018, without consideration of the reference definition of RKI. The reference definition of RKI requires the presence of symptoms and laboratory diagnosis.
